# Determination of core and shell radii of individual core–shell magnetic nanoparticles by magnetic force microscopy

**DOI:** 10.1039/d6na00233a

**Published:** 2026-06-30

**Authors:** Livia Angeloni, Luca Buccini, Pierfrancesco Atanasio, Anacleto Proietti, Ginevra Berardi, Elisabetta Carata, Luciana Dini, Mario Barteri, Francesco Mura, Davide Peddis, Mauro Pasquali, Marco Rossi, Francesca Anna Scaramuzzo, Daniele Passeri

**Affiliations:** a Department of Basic and Applied Sciences for Engineering, Sapienza University of Rome Rome Italy daniele.passeri@uniroma1.it; b Department of Chemistry, Sapienza University of Rome Rome Italy; c Dipartimento di Scienze e Tecnologie Biologiche ed Ambientali, University of Salento Lecce Italy; d Department of Biology and Biotechnologies ‘Charles Darwin’, Sapienza University of Rome Rome Italy; e Research Center for Nanotechnology Applied to Engineering of Sapienza University of Rome (CNIS) Rome Italy; f Department of Chemistry and Industrial Chemistry, Genova INSTM RU, nM2-Lab, University of Genova Genova Italy

## Abstract

The characterization of the thickness of a nonmagnetic coating is a primary issue in the investigation of core–shell magnetic nanoparticles (NPs). Here, a method based on magnetic force microscopy (MFM) is reported in which curves of the magnetic phase shift as a function of the lift height are acquired on selected NPs, corrected for electrostatic artifacts, and analyzed using a two-dipole model to describe the tip–sample magnetic interaction. After calibration of the tip on uncoated magnetic NPs, the thickness of the coating of core–shell NPs is determined. The method is demonstrated on core–shell NPs with a magnetite core and silica shell, highlighting its potential and limitations.

## Abstract 2

A method based on magnetic force microscopy is reported where magnetic phase shift *vs.* lift height curves are analyzed to determine core radius and shell thickness of core–shell magnetic NPs.

## Graphical abstract

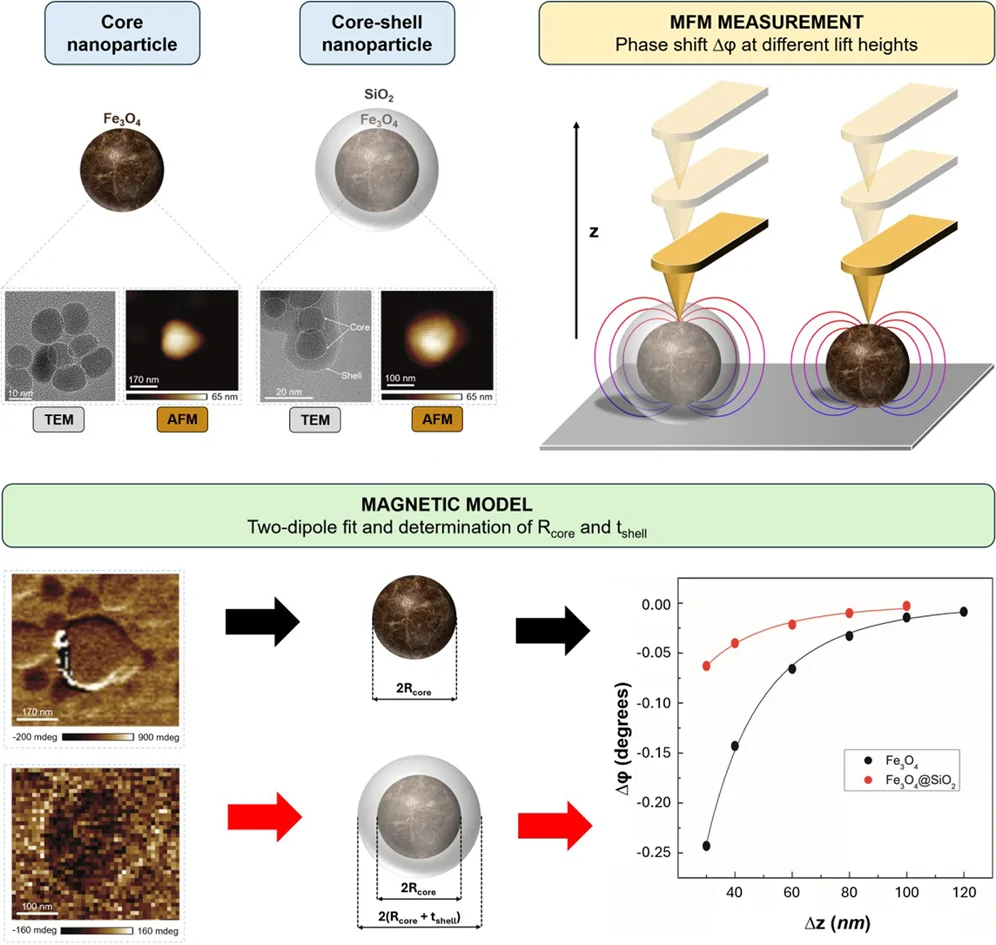

## 1 Introduction

The design, fabrication, and use of either organic or inorganic nanostructures for theragnostics is a challenge for near-future frontiers in medicine.^1–4^ Among all the possible systems, core–shell nanoparticles (NPs), which have already been used in many different fields,^5–7^ are extremely promising, as they combine the features and properties of all different components, which can even work with synergistic effects. This is particularly noticeable in core–shell systems having a magnetic core (*e.g.*, Fe_3_O_4_) covered by a nonmagnetic shell. Such architectures have been investigated to design NPs that combine the advantages of the magnetic core with tailored physico-chemical properties of the shell, *e.g.*, optical properties, enhanced biocompatibility, and possibility of being functionalized with specific molecules. For instance, magnetic NPs covered with silica (SiO_2_) can be used to combine magnetic and optical properties.^8^ Focusing exclusively on the biomedical field, magnetic NPs are extremely useful *per se*, because of their responsiveness to magnetic fields and the possibility to functionalize their surface, which can be exploited, for example, for drug delivery and imaging.^9–11^ However, the addition of a suitable external shell opens even broader prospects, giving the possibility to enhance some specific features such as biocompatibility and stability in water.^8,12,13^ When the nonmagnetic shell is a noble metal such as Au, Ag, or Cu, it is possible to obtain magnetic nanosystems that can be used in hyperthermia, for drug delivery or as theragnostic agents.^14–16^

Optimization of the synthetic procedure is fundamental to achieving perfect control and fine tuning of different morphological and physico-chemical parameters that affect the overall properties of the core–shell nanoparticles. Among these parameters, the diameter of the core and thickness of the shell have proven to be critical for their main features, and consequently for the performance of the obtainable materials.^17^ While the physical outer diameter of the NPs can be measured using different well-established techniques, *e.g.*, direct imaging of the particles using transmission electron microscopy (TEM) or analysis of the profile of NPs dispersed on flat substrates using atomic force microscopy (AFM), measuring the dimensions of the different components is much trickier. For example, Shard's methodology has been used for the conversion of XPS peak intensities into shell thickness, and the experimental determinations proved to be in good agreement with simulated spectra.^18^ However, TEM or, even better, high-resolution TEM (*i.e.*, HR-TEM) is by far the technique of choice, and has been successfully used to quantify the coating thickness on iron oxide NPs containing both magnetite and maghemite phases,^19^ investigate the crystalline structure of the core in silica-coated iron oxide NPs,^20^ and to characterize NPs with multiple cores.^21^ TEM analysis of clusters of NPs with magnetite/maghemite cores and silica shells was also successfully exploited for computational studies.^22^ TEM allows the analysis of single NPs, but measurement of the coating thickness can be non-trivial since it requires a sufficient contrast between the materials of the core and of the shell. Consequently, correlative analysis has been widely exploited, with the twofold goal of minimizing doubtful results and getting deeper insight into the system features, as for many other different nanomaterials and devices.^23–26^ For example, TEM has been coupled with alternating gradient force magnetometry (AGFM) to investigate the internal structure of superparamagnetic (SPM) NPs coated with SiO_2_, thus obtaining information on both the shell thickness and the core diameter.^8^ Briefly, magnetization curves obtained using AGFM indicated a reduction of the saturation magnetization (*M*_s_) in the presence of thicker shells due to the increase of the total amount of nonmagnetic material in the sample. Thus, the measurement of *M*_s_ could be used as an indirect method to evaluate the thickness of the nonmagnetic shell of magnetic NPs. Silica-coated iron oxide NPs have also been investigated using TEM and small-angle X-ray scattering (SAXS),^27^ while TEM coupled with magnetorelaxometry (MRX) allowed the analysis of magnetite SPM NPs coated with polyacrylic acid.^28^ Finally, AFM can be used to deduce the shell thickness from the difference between the mean diameter of uncoated and coated NPs.^29^ However, this requires statistics on a sufficiently large number of NPs, which must be rather uniform in diameter.

Recently, we proposed a different approach for the evaluation of the shell thickness based on the use of magnetic force microscopy (MFM).^30,31^ MFM is a variation of AFM in which a magnetically coated tip is used to probe magnetic fields near the surface of the investigated samples and, thus, images reflecting magnetic properties of the sample are collected simultaneously to its standard morphological reconstruction.^32,33^ Therefore, over the last three decades, MFM has been extensively used to characterize different types of magnetic materials, from the micrometer to the nanometer scale, thanks to its sensitivity to ultra-low magnetic fields, high spatial resolution, and possibility to operate in different environments (air, vacuum, liquid) and at variable temperature. Moreover, MFM allows performing experiments with no external magnetic fields or with the application of either static or dynamic external magnetic fields, nondestructively and with minimal sample preparation.^34–36^ Among the broad range of its applications, MFM has also been extensively employed for the investigation of magnetic NPs. For instance, qualitative analysis of MFM images allowed the detection of magnetic NPs in nonmagnetic matrices,^37–41^ in vesicles,^42,43^ or in biological samples like cells and tissues.^42,44–51^ On the other hand, quantitative analysis of MFM signals allowed the evaluation of the magnetic moment or the magnetization of single magnetic NPs selected on the sample surface.^52–55^ The combination of qualitative detection of magnetic NPs buried in nonmagnetic matrices and quantitative evaluation of magnetic properties of isolated NPs suggested the use of MFM to determine the actual size of the magnetic core of core–shell NPs, relying on the detection of the MFM signal as a function of the tip–sample distance. Indeed, in MFM, the tip–sample magnetic interaction is probed at a controlled distance between the tip and the sample surface. The presence of a nonmagnetic (*i.e.,* paramagnetic or diamagnetic) layer on a material with detectable magnetic moment does not produce a significant variation in the overall magnetic field, but reduces the detected magnetic field by increasing the actual distance between the tip and the magnetic sample. Thus, the thickness of the nonmagnetic layer can be inferred from the reduction of the magnetic field provided, when a suitable model for the tip–sample magnetic interaction is used.

We have already demonstrated the reliability of this method by using nonmagnetic films on periodically patterned magnetic samples.^56^ Subsequently, we proposed the extension of the method to the analysis of core–shell NPs, reporting preliminary results on Fe_3_O_4_@Cu, where the average thickness of the Cu shell determined with MFM was compared with the average difference in the diameter between uncoated and coated magnetite NPs.^29^ More recently, we used the method to estimate the thickness of the oxide/hydroxyde layer on Co-based NPs.^55,57^ Nevertheless, the validation of the method using an independent evaluation of the shell thickness is highly desirable. In this work, we fully validate our method upon comparing MFM characterization results with the direct observation of the Fe_3_O_4_@SiO_2_ core–shell NP structure by TEM. Not only are Fe_3_O_4_@SiO_2_ NPs extremely interesting for different scientific and technological applications, but they can also ensure a high contrast between the magnetite core and the silica shell in TEM imaging. All the steps of the experimental MFM method and of the subsequent data analysis are described in detail. Moreover, the results on both single NPs and small clusters are discussed, highlighting limitations, advantages, and prospects of our technique.

## 2 Materials and methods

### 2.1 Synthesis of core–shell Fe_3_O_4_@SiO_2_ NPs

#### 2.1.1 Fe_3_O_4_ core

The Fe_3_O_4_ core was synthesized by means of an easy to perform, cost effective and quick protocol developed by slightly modifying Massart's procedure.^58^ The nucleation of Fe_3_O_4_ was carried out using the co-precipitation method, adding a solution containing Fe^2+^/Fe^3+^ at molar ratio of 1 : 2 dropwise to an ammonia solution at room temperature. In a typical synthesis, 200 mL of an aqueous solution containing 3.8 mmol of (NH_4_)_2_Fe(SO_4_)_2_·6H_2_O, 3.8 mmol of Fe_2_(SO_4_)_3_ and 19.1 mmol of polyvinylpyrrolidone (PVP, MW 10000) were added dropwise to 600 mL of NH_4_OH (0.7 M) under an argon atmosphere. After this, an additional 19.1 mmol of PVP were dissolved in the mixture. The mixture was mechanically stirred for 30 minutes at room temperature, then heated at 80 °C and stirred for 30 minutes more. Once cooled, Fe_3_O_4_ MNPs were magnetically separated from the mixture by applying external permanent magnets and purified by dialysis against bi-distilled H_2_O for 24 hours. After drying under vacuum, about 700 mg of Fe_3_O_4_ MNPs were obtained. Keeping an inert atmosphere during the synthetic process is crucial to avoid the oxidation of Fe^2+^ that can lead to the formation of maghemite (γ-Fe_2_O_3_) instead of magnetite, thus modifying the magnetic properties of the MNPs. Moreover, the vigorous mechanical stirring and the use of a high molar excess of PVP allow minimization of the aggregation process normally occurring among Fe_3_O_4_ MNPs through the formation of µ-oxo bonds. Finally, the heating step promotes the loss of –OH surface groups involved in the aggregation processes, facilitating the formation of the crystal structure in Fe_3_O_4_ nuclei.

#### 2.1.2 SiO_2_ shell

The deposition of the SiO_2_ shell on Fe_3_O_4_ MNPs was carried out *via* a sol–gel process known as the Stöber method,^59^ based on the polymerization of tetraethoxysilane (TEOS) in alcohol solution using ammonia as a catalyst. The whole procedure consists of the hydrolysis of TEOS in isopropyl alcohol followed by condensation on the surface of Fe_3_O_4_ NPs, leading to the formation of the SiO_2_ shell. In a typical synthesis, 100 mg of Fe_3_O_4_ NPs were dispersed in 6 mL of bi-distilled H_2_O and sonicated for 15 minutes. Subsequently, 20 mL of a solution of TEOS in isopropyl alcohol (concentration equal to 2.5 mg mL^−1^) and 1 mL of NH_4_OH (0.7 M) were added slowly to the dispersion under mechanical stirring. The mixture was mechanically stirred for 3 hours at room temperature, then Fe_3_O_4_@SiO_2_ NPs were magnetically separated by applying external permanent magnets and washed with isopropyl alcohol and bi-distilled H_2_O. After drying overnight under vacuum, about 75 mg of Fe_3_O_4_@SiO_2_ NPs were obtained.

### 2.2 Transmission electron microscopy

The Fe_3_O_4_ and Fe_3_O_4_@SiO_2_ NPs were characterized and their size determined *via* TEM using two different instruments: (i) a Hitachi HT7700 at 120 kV, after dispersing the NP powder samples in anhydrous ethanol using an ultrasonic water bath and then depositing drops on Formvar-coated copper grids for TEM observation, and then measuring the particle size distribution using Hitachi EMIP 5.2 software; (ii) a JEOL JEM-F200 TEM in high-resolution mode (HR-TEM), operated at an accelerating voltage of 200 kV, equipped with energy dispersive X-ray (EDX) spectroscopy, after preparing the samples by depositing drops of the suspensions onto Formvar/carbon-coated copper grids, and then analyzing the NP size distribution with ImageJ software.

### 2.3 Atomic force microscopy and magnetic force microscopy

#### 2.3.1 Experimental setup

AFM and MFM experiments were conducted on samples prepared by depositing drops of the dispersed NP samples on (100) Si single-crystal substrates. Morphological analysis was performed on a commercial AFM platform (Dimension Icon, Bruker Inc.), equipped with commercial silicon probes (RTESP, Bruker Inc.) with a nominal first resonance frequency of 300 kHz and nominal spring constant *k*_c_ = 40 N m^−1^. The same platform was used for MFM analyses, using commercial silicon tips coated with a CoCr layer (MESP, Bruker Inc.), with a nominal first resonance frequency of 75 kHz and nominal spring constant *k*_c_ = 3 N m^−1^. The tips have a nominal magnetic moment *m*_tip_ = 1 × 10^−13^ emu, which was confirmed through previous MFM experiments.^54^ Experiments were conducted in air and at room conditions. The acquired MFM signal was the phase shift Δ*φ*. In order to subtract the effect of electrostatic tip–sample interaction from the standard MFM signal, we applied the method described in Angeloni *et al.*,^60^ which is similar to the differential MFM proposed by Wang *et al.*^61^. The experimental setup is equipped with an in-house-assembled electromagnet placed under the sample, capable of producing a controlled magnetic field normal to the sample surface.^62^ Using a standard floppy disk as the reference sample, the remanent magnetization curve of the magnetic tip is reconstructed, thus identifying its remanent saturation field *H*_rs_ and coercivity *H*_rc_.^60^ In our experimental procedure, a standard MFM image is first acquired with the tip fully magnetized by applying *H*_rs_. Then, −*H*_rs_ is applied in order to demagnetize the tip. In this condition, a second image of the area is acquired that reflects the tip–sample electrostatic interactions. This second image is subtracted from the standard MFM one in order to obtain an image reflecting the pure magnetic tip–sample interactions.^60^ Really, due to the discrete values of the electric current produced by the power supply, the complete demagnetization of the tip is not always achieved. Indeed, a certain residual magnetization of the tip can be observed, which is a fraction *ε* of the magnetization saturation.^60^ Although this does not affect the complete removal of electrostatic artifacts, it reduces the measured magnetic Δ*φ* signal. For accurate evaluation of the magnetic parameters, it is necessary to correct the difference signal by multiplying it by 1/(1 − *ε*) if the sample magnetization is not dependent on that of the tip; we will refer to this as ‘ferromagnetic’ (FM) correction in this work.^60^ Conversely, the difference signal must be multiplied by 1/(1 − *ε*^2^) if the sample magnetization is dependent on the magnetic field generated by the tip, as in the case of superparamagnetic NPs; we will refer to this case as ‘superparamagnetic’ (SPM) correction in this work. In the experiments described in this work, we measured a value of *ε* = 0.3.

#### 2.3.2 Theory

MFM is an AFM-based two-pass technique in which a tip coated with a magnetic layer is used to probe magnetic fields generated by the sample. During the first pass, the surface profile is acquired to reconstruct the sample morphology. The same line is scanned a second time using the profile acquired during the first pass to maintain the tip at a fixed distance from the surface, *i.e.*, the lift height Δ*z*.^33^ Indeed, if the height of the probe is kept constant during the scanning, *i.e.*, the tip is moved following a horizontal line, the actual tip–sample distance is modulated during the acquisition because of the variation in morphology of the sample due to both the inclination of the sample on the sample holder (thus the sample's morphology will be characterized by a mean plane inclined with respect to the horizontal) and the surface roughness changing the local height of the sample. As a consequence, if the tip is moved on a horizontal line (*i.e.*, in constant-height mode) the tip–sample distance would be continuously varied due to the variations in the local sample height. As the magnetic field dramatically depends on the distance, this would result in an uncontrolled variation of the magnetic field due to changes in the distance, which would dominate the contrast, producing artifacts in the MFM images. For this reason, standard MFM is generally performed in lift mode, in order to maintain a constant distance between the apex of the probe and the point of the surface of the sample under it during the imaging. However, it must also be emphasized that, while maintaining a constant lift height avoids the main artifacts due to the modulation of tip–sample distance, the modulation of the lateral morphology of the sample in the area around the projection point below the apex is responsible for morphology-induced electrostatic artifacts, which can be reduced or eliminated using our methodology, as detailed below. At sufficiently large Δ*z*, the tip–sample interaction is due to only long range forces, *i.e.*, electrostatic and magnetic. During the second pass, the cantilever is excited at (or near) its first resonance frequency and one of the oscillation parameters, which are related to the vertical gradient of the tip–sample interaction forces, is acquired. In MFM, tip–sample electrostatic interactions represent artifacts, produced by both the modulation of the tip–sample bias voltage and capacitance,^63–66^ which have to be removed to obtain the pure magnetic signal that can ultimately be quantitatively analyzed. After removal of electrostatic artifacts, magnetic interaction between the tip and a magnetic NP can be described through the simple two-dipole model,^67^ while in principle more comprehensive models should be considered for small clusters of NPs.^68^ Therefore, the dependence of the magnetic phase shift Δ*φ* on the lift height Δ*z* in the case of a core–shell NP can be modeled through the relation1
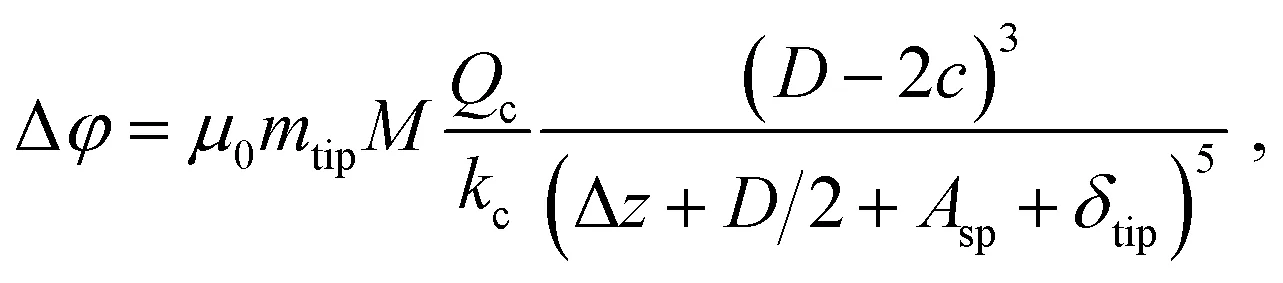
where *µ*_0_ is the free-space magnetic permeability; *m*_tip_ is the magnetic moment of the tip; *Q*_c_ is the quality factor of the first resonance of the tip; *A*_sp_ is the set-point amplitude used during the acquisition of the profile in tapping mode during the first pass; *δ*_tip_ is the distance from the tip apex of the location of the magnetic dipole describing the tip; *D* is the external diameter of the NP, given by *D* = *d* + 2*c*, where *d* is the diameter of the magnetic core and *c* the thickness of the nonmagnetic shell; and *M* is the magnetization of the magnetic core of the NP.^55^ Notably, in this work we use the generic indication of a ‘nonmagnetic’ shell referring to either paramagnetic or diamagnetic shell materials. It is clear that a paramagnetic shell would, in principle, contribute a weak attractive component to the stray field, whereas a diamagnetic shell would contribute a weak repulsive component. As the SiO_2_ shell material is diamagnetic (with susceptibility in the order of −10^−5^), a slight reduction of the attractive tip–sample interaction should be expected. However, the susceptibility-induced field is negligibly small compared to the field generated by the magnetite core. The dominant and measurable effect is thus a geometric one: the shell simply increases the physical distance between the MFM tip and the magnetic core, which is responsible for the reduction of the detected magnetic field.

## 3 Results and discussion

Typical TEM images of Fe_3_O_4_ NPs are shown in Fig. 1. In particular, Fig. 1(a) shows a typical example of a cluster of NPs. Smaller clusters where NPs are clearly distinguishable are shown in Fig. 1(b) and (c), with dotted lines added as a guide for the eyes. The distribution of the diameters of the Fe_3_O_4_ NPs, analysed for 431 NPs, is reported in Fig. 1(d), from which the diameter of the NPs can be estimated as *d* = 12 ± 3 nm.

**Fig. 1:**
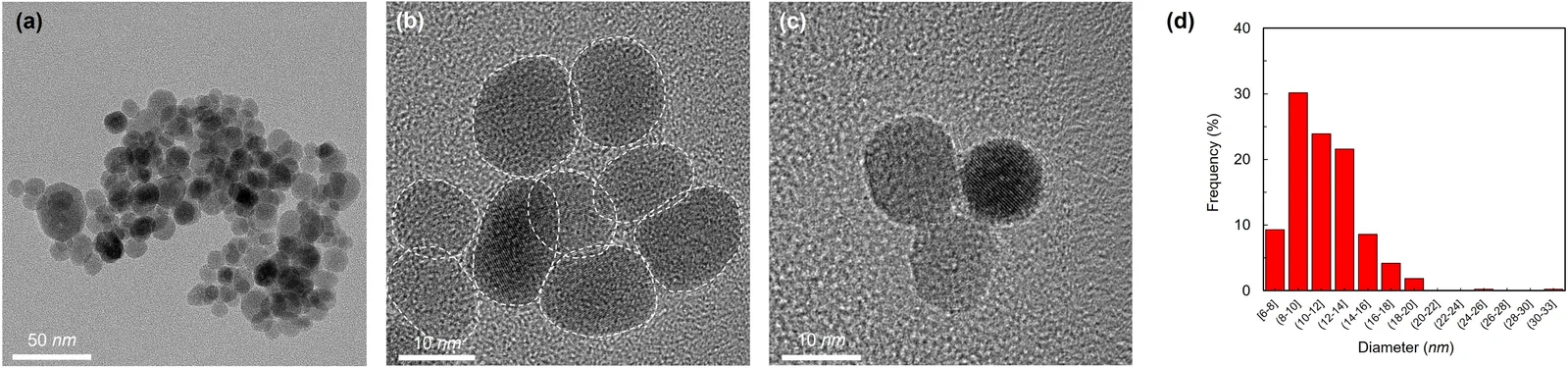
TEM characterization of Fe_3_O_4_ NPs. (a) Typical cluster of NPs. (b) and (c) Smaller clusters of NPs where single NPs are distinguishable. (d) Histogram of the distribution of the diameter of the Fe_3_O_4_ NPs, calculated for 431 NPs.

Typical TEM characterization of the Fe_3_O_4_@SiO_2_ NPs is reported in Fig. 2. In particular, Fig. 2(a) shows a cluster of Fe_3_O_4_@SiO_2_ NPs, while a higher magnification of NPs is reported in Fig. 2(b) and (c), where dotted lines are added as a guide for the eyes. A histogram of the external diameter of the core–shell particles obtained *via* 41 measurements is reported in Fig. 2(d), from which the value of *D* = 23 ± 4 nm is calculated. The histogram of the shell thickness obtained *via* 96 measurements is reported in Fig. 2(d) as well, from which the value of *c* = 7 ± 1 nm is obtained. For the sake of completeness, the diameter of the core of the Fe_3_O_4_@SiO_2_ NPs was independently measured for 77 NPs, obtaining the value of 12 ± 3 nm, confirming that the procedure of formation of the silica shell does not alter the Fe_3_O_4_ core. The detail of a single Fe_3_O_4_@SiO_2_ NP is reported in Fig. 2(e), which is correlated with the EDX elemental map of Si (f), O (g), and Fe (h), clearly showing the core–shell structure. Both Fe_3_O_4_ and the Fe_3_O_4_@SiO_2_ show the presence of agglomerated and not monodispersed NPs.

**Fig. 2:**
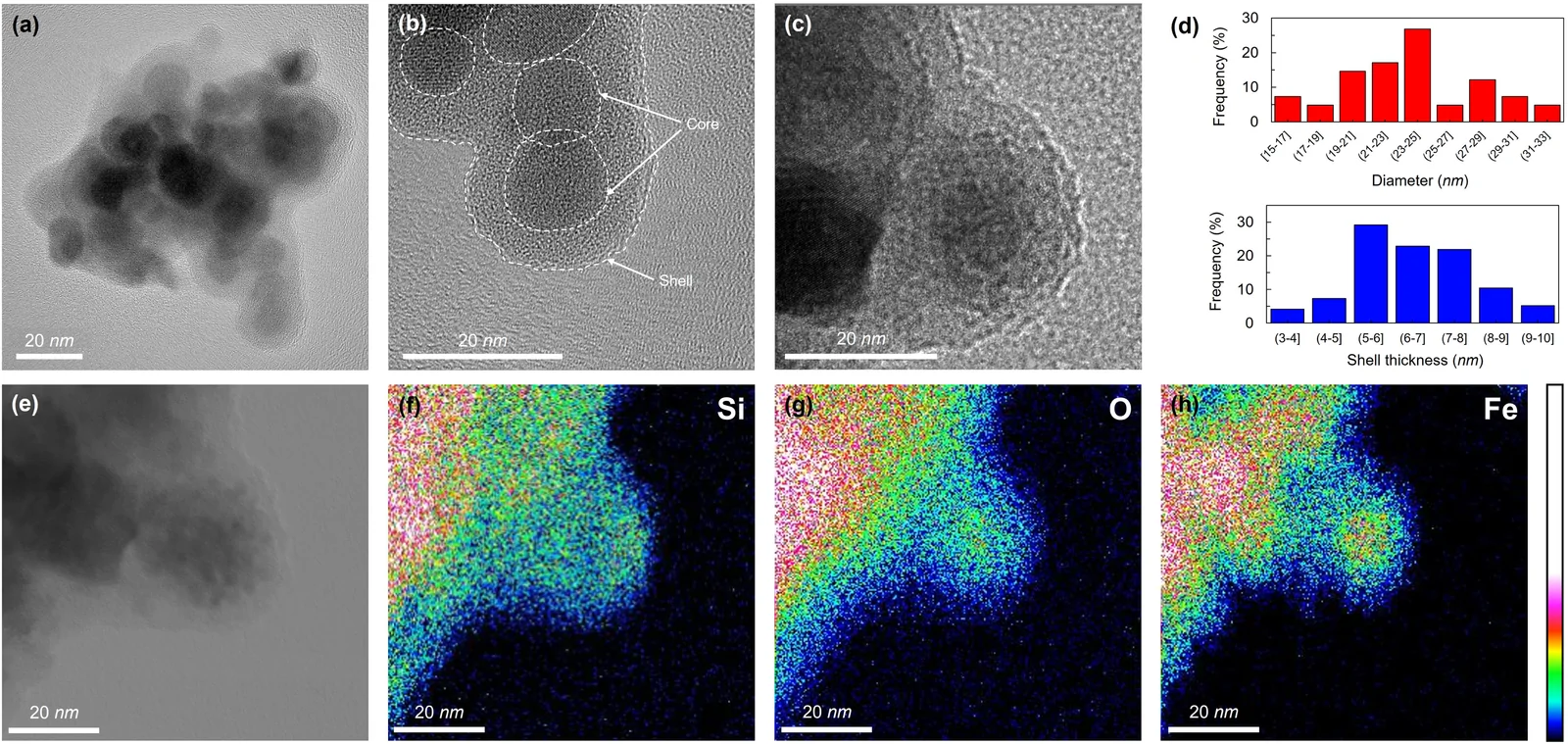
TEM characterization of Fe_3_O_4_@SiO_2_ NPs. (a) Typical cluster of NPs. (b) and (c) Details of smaller clusters of NPs where single NPs are distinguishable. (d) Histogram of the distribution of the diameter (in red) obtained for 41 NPs and of the shell thickness (in blue) obtained for 96 NPs of the Fe_3_O_4_@SiO_2_. (e) Detail of a NP with the elemental maps of (f) Si, (g) O, and (h) Fe.

An example of AFM characterization of the Fe_3_O_4_ NPs is reported in Fig. 3. An example of AFM topography is shown Fig. 3(a), where four profiles evidencing five NPs are reported. The height of the NPs (calculated as the difference between the top of the NP and the plane) is reported, which is used as the measurement of the diameter of the NP. Notably, the diameter of the NPs is not estimated using the ‘in-plane’ diameter as the value is increased because of the apparent geometrical dilation of the NPs due to the tip–sample convolution effect.^69^ The diameters of the imaged NPs range between 14 nm (which can be rationalized as a single NP) to 40 nm (which is likely to be a small cluster of NPs). Statistical analysis of the diameters, performed on 35 NPs, is reported in Fig. 3(b), which indicates that AFM seems to overestimate the size of the NPs. This is due to the fact that the NPs in isolated small agglomerates are indistinguishable from single NPs due to the already mentioned effect of morphological dilation on lateral dimensions in AFM images and, thus, small agglomerates are likely to be included in the statistical analysis of AFM images in addition to isolated NPs.

**Fig. 3:**
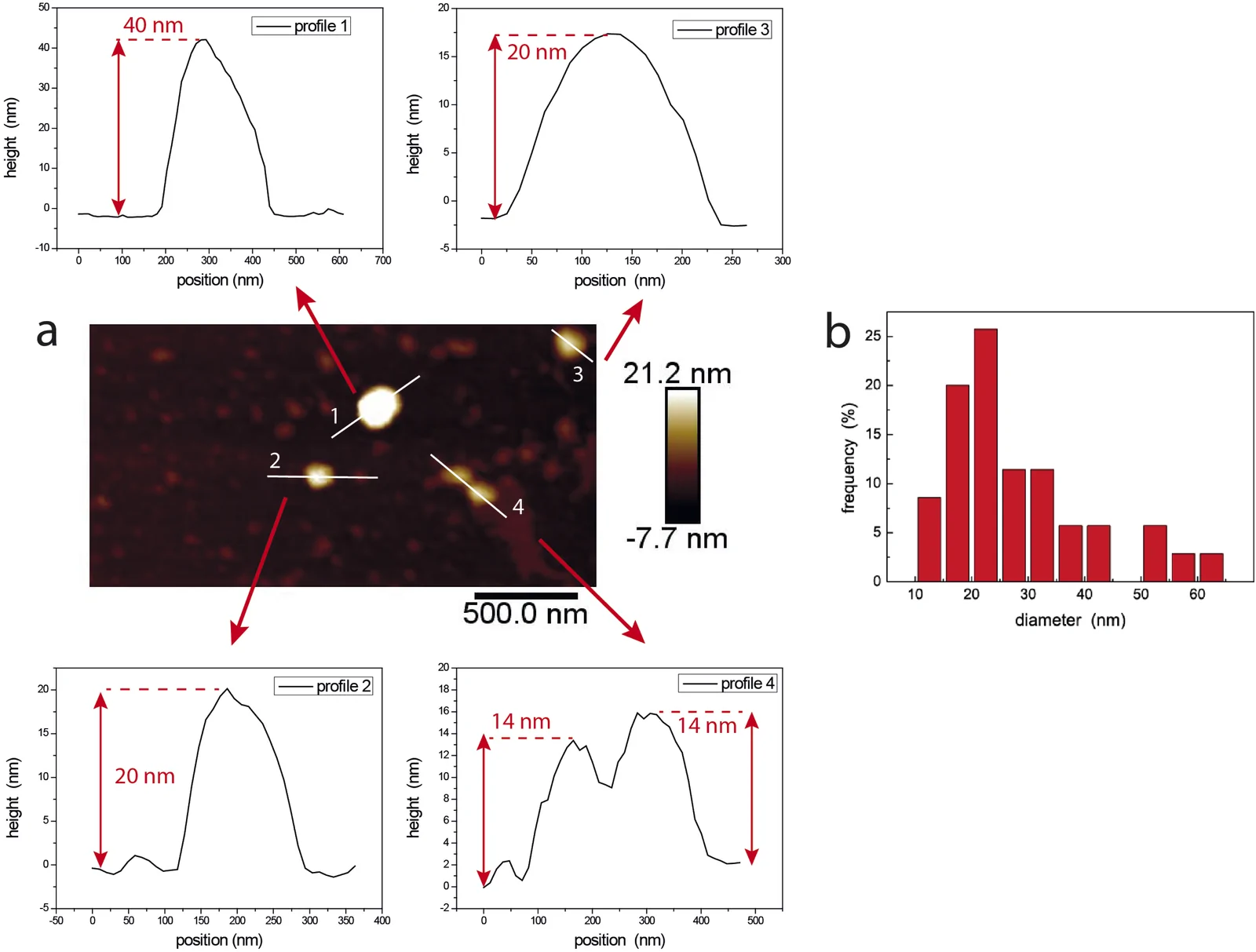
AFM characterization of Fe_3_O_4_ NPs. (a) Typical AFM morphological image, with four profiles of NPs with the height of the NPs indicated, which is used to evaluate their diameter. (b) Statistics of the diameter of Fe_3_O_4_ NPs measured through AFM morphological analysis on 35 NPs.

The MFM characterization method has been demonstrated by selecting different isolated NPs (or NP agglomerates) and measuring the MFM phase shift, at different lift heights, corresponding to the center of the NPs. The data analysis procedure is summarized in Fig. 4.

**Fig. 4:**
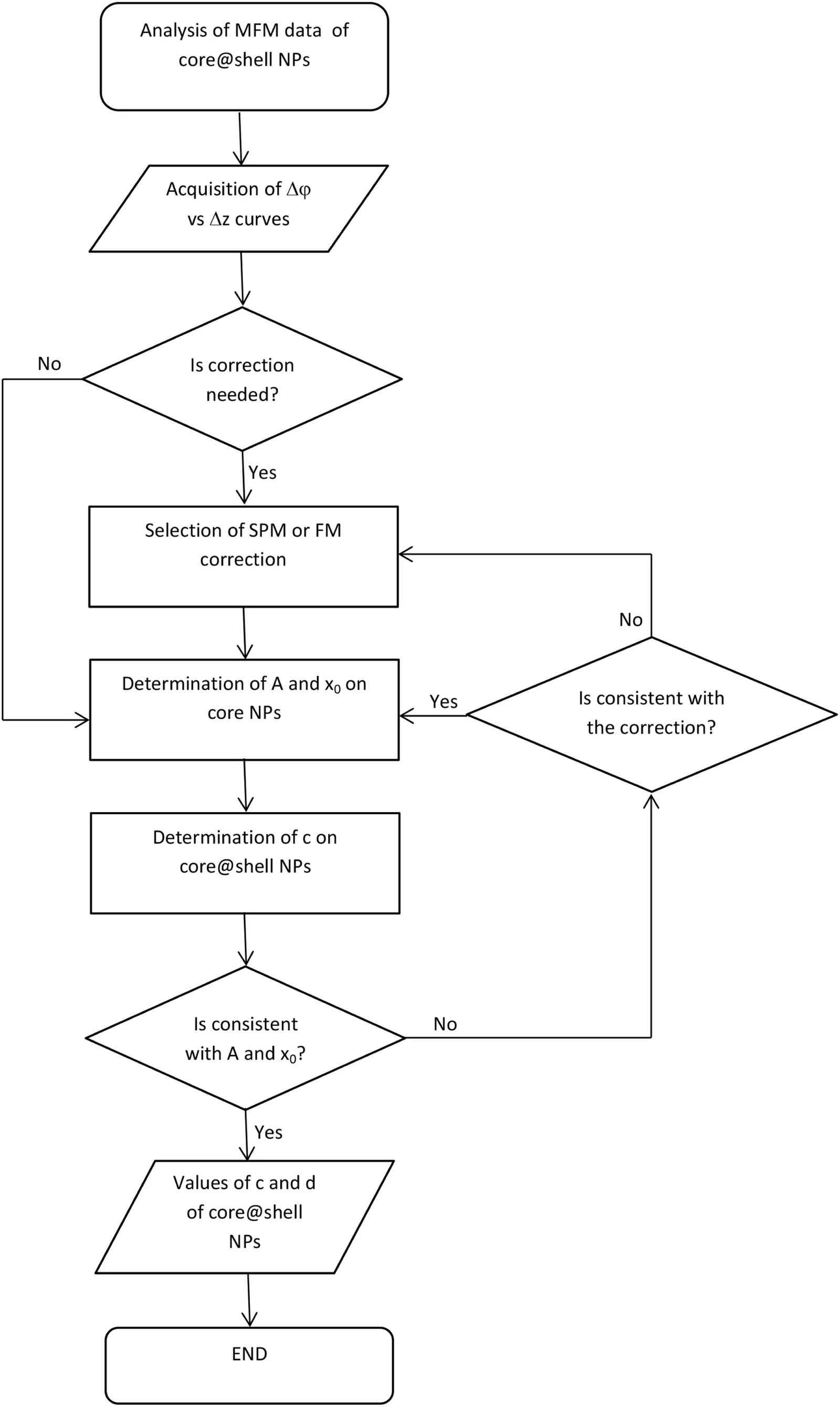
Flux diagram summarizing the data analysis procedure to evaluate the shell thickness of core–shell NPs from MFM measurements.

In order to illustrate the method, examples of MFM imaging of magnetic NPs are shown in Fig. 5. Simultaneously to the morphological reconstruction of the surface of the Fe_3_O_4_ NPs sample, reported in Fig. 5(a), the standard MFM signal obtained from the second-pass cantilever phase shift is acquired, which is shown in Fig. 5(b). After demagnetization of the probe, the image of the electrostatic signal is acquired (Fig. 5(c)), which is ultimately subtracted from the standard MFM signal in order to obtain the image of the pure magnetic signal, reported in Fig. 5(d). An example of the same characterization performed on the Fe_3_O_4_SiO_2_ NP sample is reported in Fig. 5(e) (topography), Fig. 5(f) (standard MFM image), Fig. 5(g) (phase image with the demagnetized probe), and Fig. 5(h) (subtraction of the latter two images). An example of MFM analysis of single NPs is shown in Fig. 6(a).

**Fig. 5:**
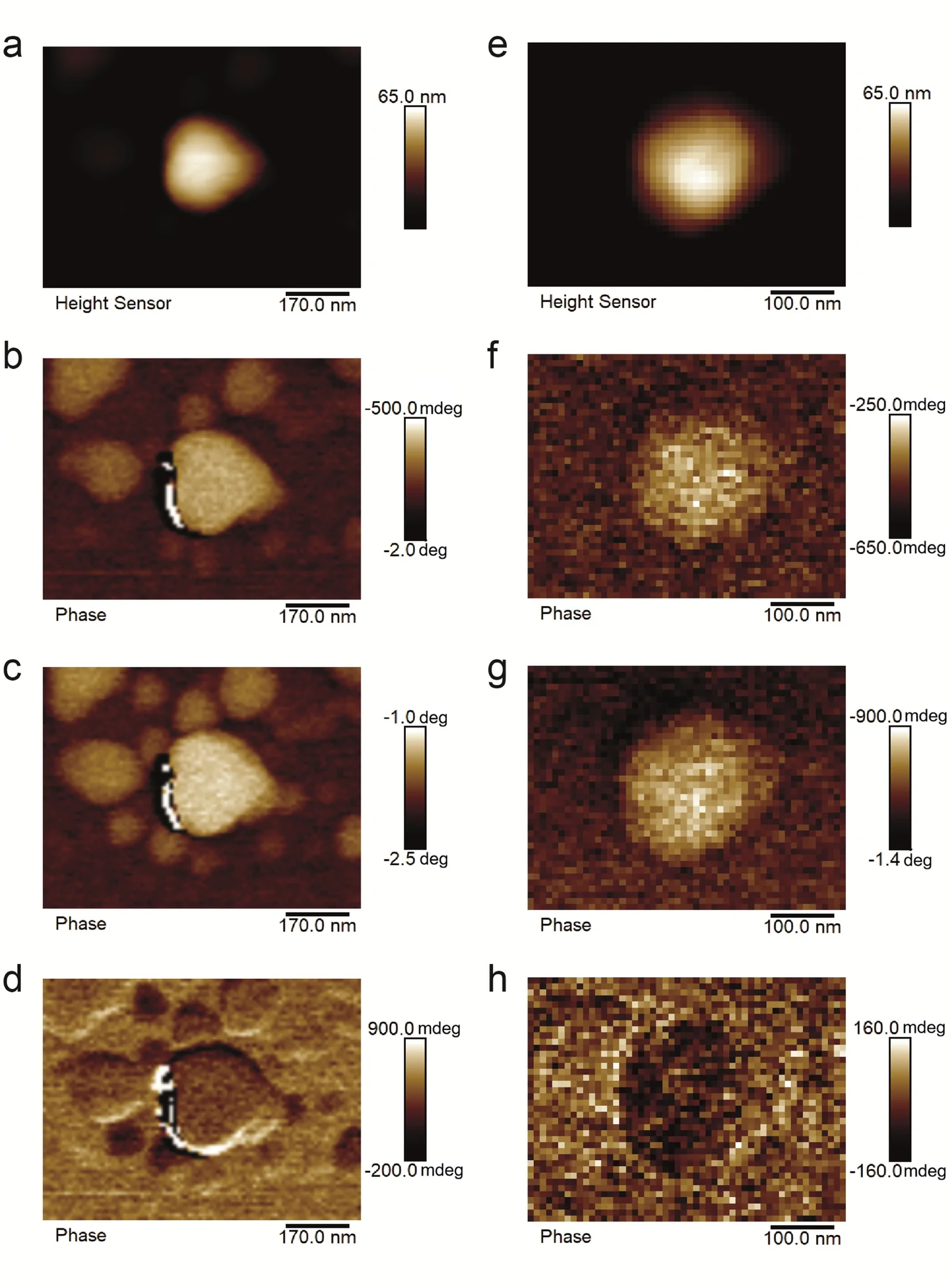
Example of MFM characterization of an Fe_3_O_4_ NP: (a) morphological image, (b) standard MFM phase image, (c) electrostatic signal, and (d) pure magnetic signal. Example of MFM characterization of an Fe_3_O_4_@SiO_2_ NP: (e) morphological image, (f) standard MFM phase image, (g) electrostatic signal, and (h) pure magnetic signal.

**Fig. 6:**
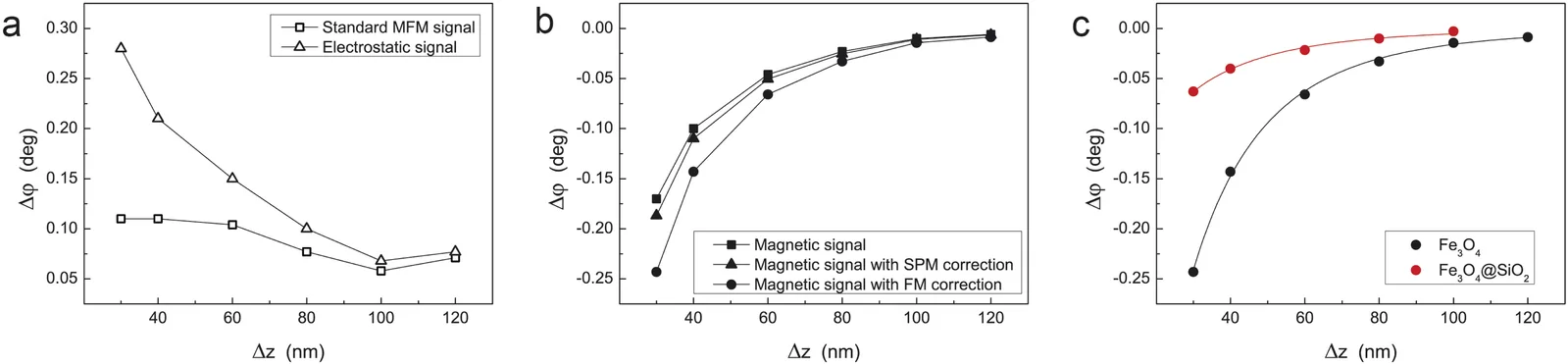
Example of MFM analysis of an Fe_3_O_4_ NP with a diameter of 41.2 nm: (a) values of phase shift Δ*φ* corresponding to the standard MFM signal (open squares) and electrostatic signal (open triangles) as a function of the lift height Δ*z*. (b) Values of Δ*φ* as a function of Δ*z* corresponding to the pure magnetic signal obtained by subtracting the MFM and electrostatic signals (squares) and difference signal after SPM correction (triangles) and FM correction (circles). (c) Magnetic Δ*φ* as a function of Δ*z* obtained on an Fe_3_O_4_ NP with diameter of 41.2 nm (black circles) and on an Fe_3_O_4_@SiO_2_ NP with diameter of 63.3 nm (red circles), both after FM correction.

The standard MFM phase shift Δ*φ* measured as a function of the lift height Δ*z* for an Fe_3_O_4_ NP with a diameter of 41.2 nm, reported in Fig. 6(a) (open squares), does not correspond to any simply explainable tip–sample interaction. Indeed, positive values of Δ*φ* are observed, which would indicate a repulsive tip–sample magnetic force. This, however, is not compatible with the possible scenarios expected in our experiments. First, it must be observed that a positive value of Δ*φ* in the standard MFM signal indicates a repulsive tip–sample interaction, while a negative value of Δ*φ* indicates an attractive one. As the magnetic field from a magnetic dipole decreases with distance (*i.e.*, with the reciprocal of the cube of the distance), the magnetic force on the probe decreases. As the phase shift is proportional to the gradient of the magnetic force, it is expected to decrease to zero as the distance increases. Really, the relation between the phase shift and the distance should be that reported in eqn (1), *i.e.*, proportional to the reciprocal of the fifth power of the distance. However, this is the behavior expected for the pure magnetic signal. On the basis of the actual experimental configuration, the magnetization of the tip and of the sample are parallel, leading to an attractive interaction and, thus, a negative phase shift is expected. The standard MFM signal, however, is the superimposition between the pure magnetic signal, which is attractive, and the electrostatic signal (arising from the work function difference, local charge heterogeneities, and modulation of the tip–sample capacitance due to morphological variations of the sample), which is repulsive. Due to the low magnetic field produced by the NPs, the repulsive electrostatic interaction dominates the standard MFM signal. Thus, the overall dependence of the standard MFM signal on the distance cannot be easily rationalized as it is the result of different tip–sample interactions with different, and not easily describable with suitable analytical models, dependency on the distance.

This, however, is precisely the motivation for the two-acquisition protocol described in our work. As MFM is performed in the absence of external magnetic fields, if the NP is small enough to show superparamagnetic behavior, it will be oriented by the magnetic field generated by the tip and, thus, the tip–sample interaction will be attractive. Conversely, if the NP is ferromagnetic (which is the expected case considering the NP diameter), it will be at its remanent magnetization as a result of the application of *H*_rs_. Thus, the remanent magnetization of the NP and of the tip will have the same orientation and, thus, their interaction will be attractive. An additional deviance from any expected interaction model is that Δ*φ* is constant for values of Δ*z* from 30 nm to 60 nm and decreases only below this range. Values of Δ*φ* obtained after demagnetization of the tip are shown in Fig. 6(a) (open triangles) and definitely indicate a decrease in the electrostatic tip–sample interaction as Δ*z* increases. By subtracting the standard MFM signal from the electrostatic one, the Δ*φ* values corresponding to the pure magnetic signal are obtained, and are shown in Fig. 6(b) (squares). As expected, this difference signal is negative, indicating an attractive tip–sample interaction, and definitely shows a decrease as Δ*z* increases, in accordance with the model described by eqn (1).

As the residual tip magnetization when *H*_rc_ is not negligible (*ε* = 0.3), suitable correction is required to accurately evaluate the magnetic properties of the NPs. The corresponding curves obtained by multiplying by 1/(1 − *ε*^2^) (SPM correction, assuming the NP is superparamagnetic) and 1/(1 − *ε*) (FM correction, assuming the NP is ferromagnetic) are reported in Fig. 6(a) (triangles and circles, respectively). Once the suitable correction model is assumed and data are corrected, for calibration purposes, Δ*φ versus* Δ*z* values obtained for Fe_3_O_4_ NPs are analyzed using the model described by eqn (1), *i.e.*, curves are fitted using the equation2
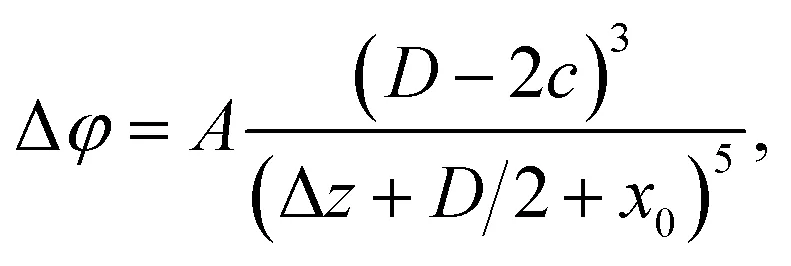
where *c* = 0 is imposed due to the absence of a nonmagnetic shell (thus *D* = *d*), while

3 *x*_0_ = *A*_sp_ + *δ*_tip_

4
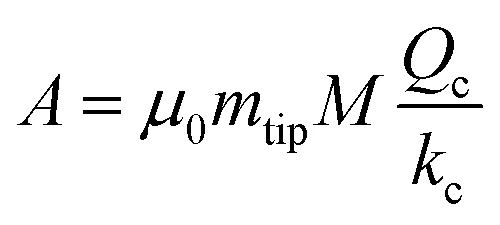
are used as fitting parameters. Notably, even if considered a fitting parameter, *x*_0_ is the sum of (i) *A*_sp_, which is the tapping set-point amplitude used during the first pass and increases the actual tip–sample distance with respect to the nominal lift height, and (ii) *δ*_tip_, the distance from the tip apex to the effective location of the magnetic dipole representing the tip. Therefore, *x*_0_ accounts for the offset between the nominal lift height Δ*z* (measured from the mean tip position during tapping) and the actual distance between the magnetic dipole describing the tip and the surface of the top of the imaged NP.

In this work, 6 different Fe_3_O_4_ NPs with diameters in the range of 21–42 nm were analyzed for calibration purposes. Δ*φ vs.* Δ*z* curves were initially corrected assuming the FM model, *i.e.*, we supposed that the NPs magnetized at their remanent magnetization independently from the magnetic field generated by the tip. Thus, the values of *A* and *x*_0_ were determined for each NP. An example of data fitting, obtained on an Fe_3_O_4_ NP with diameter 41.2 nm, is shown in Fig. 6(c) (black circles). It is worth noting that the calculation of *x*_0_ is needed to calibrate the tip, *i.e.*, to determine the position of the magnetic dipole equivalent to the tip. Conversely, according to eqn (4), the calculation of *A* can be used to determine either *m*_tip_ (assuming the value of *M* of the sample known) or *M* of the sample (assuming *m*_tip_ is known). In this work, we chose the latter approach, as while the dimension of the single Fe_3_O_4_ NP suggests a superparamagnetic behavior, bigger aggregates could show a ferromagnetic one, which results in an uncertainty in the value of *M*. Really, the obtained values of *x*_0_ are rather independent from the NP diameter and the average value *x*_0_ = 49 ± 3 nm is obtained. Conversely, while the obtained values of *A* are rather independent from the diameter in the case of bigger NPs, they are found to be significantly higher in the case of the two smaller NPs.

This result can be rationalized by calculating the corresponding values of *M* using eqn (4), considering the density of magnetite *ρ* = 5.2 g cm^−3^, and these are shown in Fig. 7(a) as a function of the diameter *d* of the NPs (black symbols). The biggest NPs with diameters ranging from 34 nm to 42 nm, indicated with an arrow in Fig. 7(a), show values of *M* independent from their diameter and the calculated average value *M* = 4.3 ± 0.2 emu g^−1^ is in good agreement with the values of remanent magnetization reported in literature for magnetite NPs in the same range of diameters.^70^ This result is consistent with the assumed FM correction model. Conversely, the two smaller NPs with diameters 21.6 nm and 29 nm show higher values of *M*, *i.e.*, 18 emu g^−1^ and 12 emu g^−1^. These are not compatible with NPs in a remanent magnetization state independent from the magnetic field generated by the tip, as for smaller NPs the remanent magnetization would rather decrease.^70^ Thus, this result indicates that these two NPs are superparamagnetic. With the observed value of *M* being far below the expected saturation magnetization of about 60 emu g^−1^,^70,71^ they are in the linear region of the magnetization curve, *i.e.*, where the NP magnetization is proportional to the tip magnetic field. This indicates that the magnetic field generated by the tip is not sufficient to saturate the NP magnetization. Therefore, while the FM correction is suitable for the biggest NPs, the SPM correction model must be used for the two smaller NPs. Repeating the data analysis starting from the Δ*φ vs.* Δ*z* values corrected after multiplication by 1/(1 − *ε*^2^), the two values of 14 emu g^−1^ and 10 emu g^−1^ are obtained for the NPs with diameters of 29 nm and 21.6 nm, respectively (green symbols). For the sake of completeness, the values of *M* obtained with the SPM correction for the biggest NPs are also reported in Fig. 7(a). Although the obtained values are lower than those obtained with the FM correction (indeed, the mean *M* value is 3.4 ± 0.1 emu g^−1^), they are again almost independent from the NP diameter, suggesting that they are actually in their remanent state. This in agreement with the results reported by Pérez *et al.*^72^ that aggregates of superparamagnetic magnetite NPs with a diameter of 16 nm are in a blocked magnetic state at room temperature when the NPs are at small interparticle spacing. This confirms the correctness of the data analysis using the FM correction model for the biggest NPs in the diameter range 34–42 nm and the SPM correction model for the smallest NP of the set, with a diameter of 21.6 nm. Finally, although the accuracy of the method is not sufficient to definitely assess the presence of a trend in the values of *M* for the bigger NPs, it is interesting to observe a slight increase in the values of remanent magnetization *M* as *d* increases, which would be in agreement with results reported by Li *et al.*^70^

**Fig. 7:**
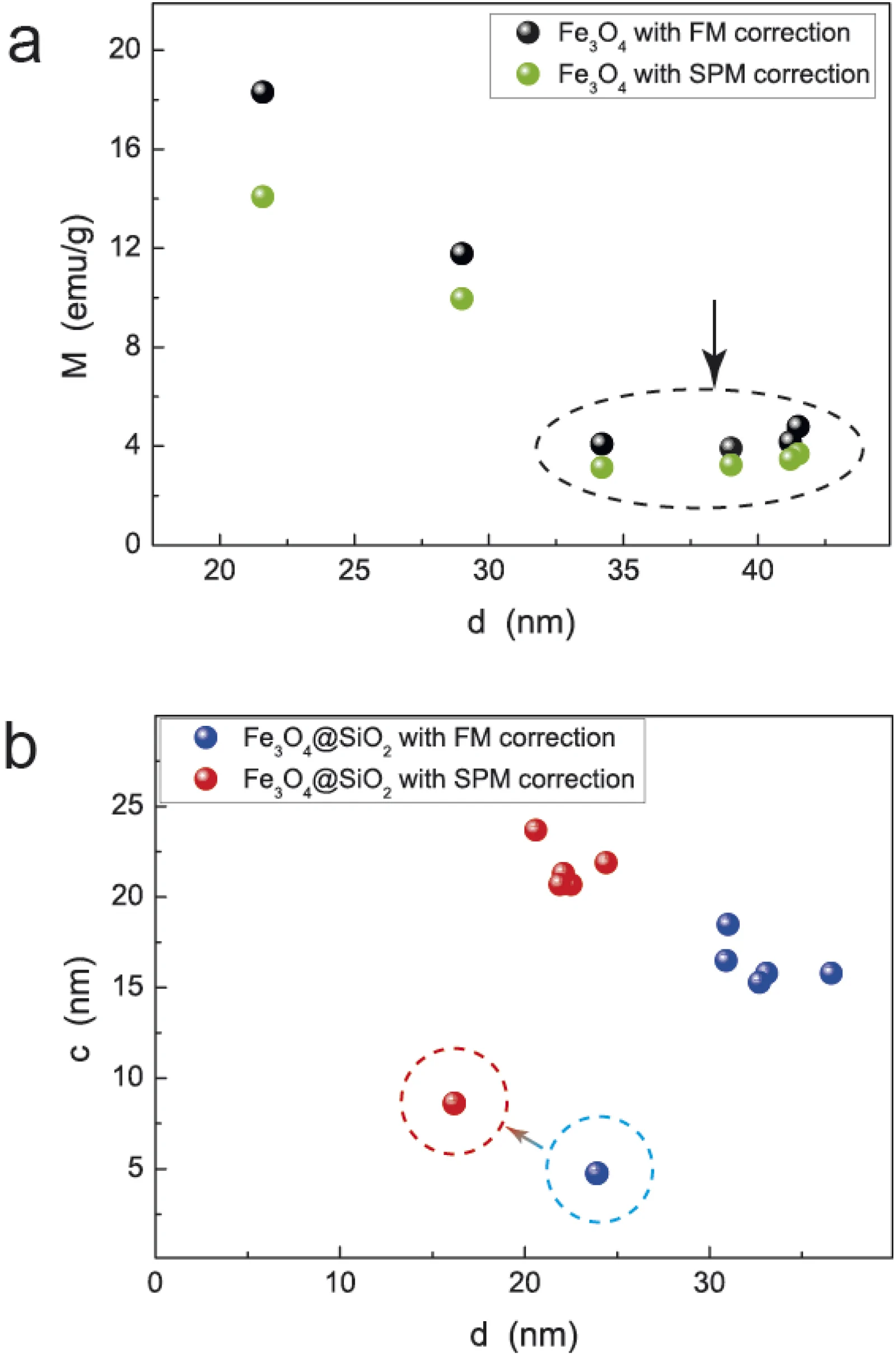
(a) Calculated values of the magnetization *M* of the Fe_3_O_4_ NPs as a function of their diameter *d*, obtained using the FM model (black symbols) or the SPM one (green symbols). (b) Calculated values of the shell thickness *c* of the Fe_3_O_4_@SiO_2_ NPs as a function of the diameter of the magnetite core *d*, obtained using the FM model (blue symbols) or the SPM one (red symbols). Data for which the FM model is incorrect are indicated with the blue dashed circle and the corresponding correct data using the SPM model are indicated with the red dashed circle.

After the calibration step, the analysis of the core–shell NPs was performed. For this purpose, a set of seven Fe_3_O_4_@SiO_2_ NPs was selected, two with an external diameter *D* of about 33 nm and five with diameters ranging from 63 nm to 68 nm. Considering the observed values of diameter, first Δ*φ vs.* Δ*z* data were corrected assuming the FM correction model. The obtained corrected data were fitted using eqn (2), assuming *x*_0_ = 49 nm and *M* = 4.3 emu g^−1^ and using *c* as the free fitting parameter. An example of the fitting of data measured on an Fe_3_O_4_@SiO_2_ NP with a diameter of 63.3 nm is shown in Fig. 6(c) (red circles). The obtained values of shell thickness *c* are reported in Fig. 7(b) as a function of the diameter *d* of the magnetic core (blue symbols). Note that while in Fig. 7(a) the diameter *d* is directly measured from AFM/MFM topography, as illustrated in Fig. 3, in Fig. 7(d)*d* is the diameter of the magnetic core of the core–shell NPs and it is calculated from the fitting of MFM curves. As expected, considering that the values of their external diameter vary in a narrow range, the values of *c* obtained for the five biggest NPs are similar and their mean value is 16.4 ± 0.6 nm and, consequently, the obtained mean value of *d* is as high as 32.9 ± 1.0 nm. On the basis of data reported in Fig. 7(b), the obtained values of *d* suggest that the cores of the biggest NPs are ferromagnetic and, thus, the parameters used in the data analysis are consistent. As for the two NPs with *D* about 33 nm, values of *c* and *d* of about 5 nm and 23 nm are obtained, respectively, corresponding to the data in the dashed circle in Fig. 7(b). Nevertheless, the value of *d* is not consistent with the initial assumption of a ferromagnetic core and, thus, of the use of the FM correction model. Indeed, on the basis of the results reported in Fig. 7(a), the SPM correction must be used.

After proper correction, Δ*φ vs.* Δ*z* data for the two smallest Fe_3_O_4_@SiO_2_ NPs were fitted using the values *x*_0_ = 55 nm and *M* = 14 emu g^−1^. From this second data analysis, the value *c* = 8.6 ± 0.1 nm is obtained for both the NPs and, thus, *d* = 16 nm for the Fe_3_O_4_ magnetic core corresponding to the data reported in Fig. 7(b) (red symbols). These values are definitely consistent with the assumed model and parameters and, thus, can be considered as the correct output corresponding to the two smallest NPs of our MFM method. Notably, the value *c* = 8.6 ± 0.1 nm retrieved with MFM characterization is in good agreement with the value *c* = 7 ± 1 nm obtained with TEM analysis. Finally, we can conclude that the Fe_3_O_4_@SiO_2_ NPs with external diameter *D* = 33 nm are constituted by a magnetite core with diameter of 16 nm and a silica shell with thickness of 8.6 nm. Conversely, the biggest Fe_3_O_4_@SiO_2_ NPs with mean external diameter *D* = 66 nm seem to be constituted by a magnetite core with diameter of 33 nm and a silica shell with thickness of 16 nm. It must be noted that, on the basis of TEM results, a NP with *d* = 33 nm can hardly be constituted by a single Fe_3_O_4_ NP. Also, the value *c* = 16 nm is much bigger than the shell thickness of about 7 nm for a single NP. Therefore, the NPs with average external diameter *D* = 66 nm are actually constituted by the aggregation of core–shell NPs. Of course, to definitely assess that these big NPs are aggregated, a correlative TEM/AFM approach would be useful. However, TEM analysis of the bare Fe_3_O_4_ NPs shows a mean diameter of 12 nm, making it physically implausible that a single Fe_3_O_4_ NP could have a core diameter of 33 nm as retrieved by our model. Also, TEM images of the Fe_3_O_4_@SiO_2_ sample show clear aggregation even before deposition on the substrate. Finally, the retrieved shell thickness is approximately double the TEM-measured individual NP shell thickness, which can hardly be supposed to correspond to a single NP, while it is qualitatively consistent with a small aggregate of core–shell NPs. Note also that, as the clusters on the TEM grid are disordered and irregular, a meaningful measurement of an ‘effective aggregate diameter’ from TEM is not straightforward. Also, it must be considered that when performing AFM/MFM, we selected the smaller and ‘more regular’ aggregates, which appeared at least roughly spherical (note that a clear distinction of the NPs of small aggregates can be difficult due to the tip-shape convolution effect). Thus, statistics on agglomerate size cannot be easily performed, either with TEM and AFM/MFM. In the case of multi-core aggregates, the single-core dipole model may be no longer strictly valid. In that case, the retrieved values must be interpreted as ‘effective’ parameters (*d*_eff_, *c*_eff_) of an equivalent single-core NP producing the same magnetic response. For the sake of completeness, the values of *d* and *c* obtained for the biggest Fe_3_O_4_@SiO_2_ NPs using the SPM correction model and the calibration performed on the 21.6 nm Fe_3_O_4_ NP have been also reported in Fig. 7(b), which correspond to the (effective) average values *d* = 22.3 ± 0.6 nm and *c* = 21.7 ± 0.5 nm.

To better understand the meaning of *d*_eff_ and *c*_eff_, we can consider a simple case. Let us suppose a small aggregate of 4 core–shell NPs disposed as a tetrahedron with a triangular base formed by 3 NPs with the fourth NP on top of them. Suppose each NP has a core with diameter *d* = 12 nm with a shell of thickness *c* = 7 nm. The outer diameter of the particle is thus *D* = 26 nm. The height of the tetrahedron (which would be considered the diameter of the cluster in AFM/MFM) would be 47 nm. The total volume of the magnetic cores is *V*_magn_ = 4 × 4/3π(*d*/2)^3^. If this volume is considered that of an equivalent sphere in the center of the NP (‘equivalent core’), the equivalent core diameter would be 
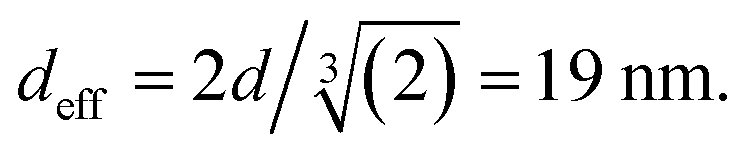
 Thus, the equivalent thickness of the shell would be *c*_eff_ = (*D* − *d*)/2 = 14 nm. Analogous calculation on a more complex cluster, *i.e.*, a cluster of 16 NPs with an icosahedral structure, would give an external diameter *D* = 66 nm, an equivalent diameter of the magnetic core of *d*_eff_ = 30 nm and an equivalent shell thickness *c*_eff_ = 18 nm. Although the considered structures are extremely simple and too regular to effectively represent the clusters of Fe_3_O_4_@SiO_2_, the calculated parameters seem meaningful when compared to the results reported in Fig. 7(b). Notably, aggregates of Fe_3_O_4_@SiO_2_ NPs can in principle show superparamagnetic behavior provided that the interparticle spacing due to the silica shell is sufficiently large to prevent dipole–dipole interaction between particles.^72^ Pérez *et al.*^72^ observed such a behavior at room temperature for magnetite NPs with a diameter of 16 nm with a silica shell 30 nm thick, while the same magnetite NPs showed a blocked magnetic state with a few-nanometer-thick oleic acid coating. Despite the absence of an independent characterization, we assume that the about 8 nm thick silica coating is not sufficient to prevent dipole–dipole interactions in small aggregates. Therefore, while the SPM model is correct for the smallest Fe_3_O_4_@SiO_2_ NPs with *D* = 33 nm, the FM model is the most suitable one for the big NPs with *D* = 66 nm, leading to the effective values of 33 nm and 16 nm for the core diameter and the shell thickness, respectively. Indeed, in aggregated systems, the reduction of the average interparticle distance strongly enhances interparticle magnetic interactions, leading to an increase in the effective energy barrier for magnetization reversal and, consequently, to a significant enhancement of the blocking temperature.^73–75^ In the present case, since the investigated systems are oxide nanoparticles separated by surfactant and/or oxide interfaces, direct exchange interactions are expected to be largely suppressed, while dipolar interactions are expected to dominate the collective magnetic response.^76,77^ Under these conditions, the system progressively deviates from the ideal non-interacting SPM regime and may exhibit a ferromagnetic-like behavior on the experimental timescale.^78^

Really, a certain ambiguity of our technique in the analysis of small aggregates cannot be ignored as only effective parameters can be obtained for such samples. Also, the ability to retrieve ‘effective’ values of the diameter of the core represents a main limitation in the case of core–shell NPs with multiple magnetic cores in each NP.^21,79^ Indeed, from the fitting of MFM curves, the diameter of the magnetic core was determined assuming, for instance, FM correction. If this value is in the range in which FM behavior is expected, the analysis is considered consistent; otherwise, data are re-analyzed with the SPM correction. Thus, this self-consistency criterion does not require knowledge of the core size as an input. Really, if the NP diameter is around the transition between FM and SPM regimes and thus the attribution to a specific regime cannot be definitely assessed, one could also assign the mean value between those obtained in the FM and SPM models and the semidifference as the uncertainty. In our work, as far as the value of *M* for the bare NPs is considered, this would lead to an uncertainty of 10–13%. As for the core–shell NPs, the uncertainty on the coating thickness would be 19% on the biggest NPs and 25% on the smaller ones. Nevertheless, although the study of the interactions among magnetic NPs in small aggregates is an issue of high scientific and technological relevance, in particular considering the applications of SPM NPs,^80^ this far exceeds the scope of this work, which is focused on the demonstration of the use of MFM for the evaluation of the shell thickness of single NPs. Notably, however, MFM has been already proposed for the analysis of SPM *versus* blocked states in small aggregates of magnetite NPs,^81^ and our technique could represent a useful tool to address this issue.

## 4 Limits and perspectives

The method we proposed undoubtedly suffers from the introduction of a certain number of approximations and assumptions, especially considering that well-established alternative methods already exist. The most valid alternative is of course TEM. Nevertheless, accessing a TEM is not straightforward. Indeed, TEM is more expensive, less versatile and general-purpose, and has higher maintenance costs than AFM, which, therefore, is an instrumentation more widespread and commonly accessible than TEM. Practically all AFM setups implement MFM, which therefore would allow our method to be implemented in existing AFM setups. Also, it must be considered that, differently from TEM, our method can also be applied on samples where core–shell magnetic NPs are grown directly on substrates, *e.g.*, NPs electrodeposited on electrodes;^55^ on this type of sample, TEM cannot be performed unless a destructive and time-consuming sample preparation is carried out, while SEM can be used to evaluate the outer diameter of the NPs but the internal structure cannot be investigated, and thus AFM–MFM represents the only method capable of giving information about the internal structure, *i.e.*, on the size of the magnetic core and the nonmagnetic shell. Finally, with some adjustment in the model, our method could also be applied to the investigation of magnetic NPs buried in paramagnetic or diamagnetic matrices.^82,83^ Also, in a future development, our method could be automatized: instead of acquiring subsequent images of the same area with the magnetized and demagnetized probe, MFM phase curves as a function of the lift height could be acquired with the magnetized and demagnetized tip. Thus, the AFM–MFM system could automatically acquire the curves, and subtract and fit them, returning as the output the thickness of the shell coating or the magnetization of the core depending on the parameter of interest.

Another advantage of the method presented is that, in principle, it is a versatile approach that can be used to characterize different physical parameters of core-only and core–shell magnetic NPs. More specifically, the parameter *A*, which is experimentally determined, contains (not independently) the magnetization of the sample *M*, the parameters of the cantilever, namely its quality factor *Q*_c_ and its spring constant *k*_c_, and the magnetic moment of the tip *m*_tip_. Therefore, depending on which parameters are assumed known, the method can be used to determine the remaining parameters. In cases where the nonmagnetic shell is absent (core-only NPs) or its thickness can be neglected, *m*_tip_ can be independently measured or assumed to be the one supplied by the manufacturer. In this case, the magnetization of single isolated NPs can be measured. This is the case for the results on Fe_3_O_4_ reported here. Conversely, if the shell can be neglected and the magnetization of the NPs is known, the same calibration procedure can be used to determine the value of *m*_tip_, analogously to what was reported by Wu *et al.*^84^. In the case of core–shell NPs, if the shell thickness is known, the magnetization of the core or *m*_tip_ can be measured, analogously to the case of core-only NPs. In the case of core–shell NPs, if the shell thickness is not known and needs to be determined (as in the case of our work), it can be evaluated by fitting MFM curves assuming the value of *x*_0_ and *A* measured in the calibration step, which is the procedure we followed in our work. In the case of core–shell NPs, it must be noted that after the calibration step, NPs with different magnetic cores can be analyzed by replacing the calibrated value of *A* (namely *A*_cal_) with the actual one *A* = *A*_cal_*M*_core_/*M*_cal_, where *M*_cal_ and *M*_core_ are the magnetization of the NPs used for calibration and of the actual material forming the core, respectively. Of course, this approach can be tricky as it requires the independent knowledge of *M*_core_. In principle, one could think of analyzing MFM curves on core–shell NPs without the calibration step, independently measuring the parameters in eqn (3) and (4) or assuming their nominal values. Due to the tip-to-tip variability, using the tip’s nominal values would highly increase the uncertainty of the method. Conversely, the independent calibration of each parameter would require different steps of calibration. Besides the value of *m*_tip_, *k*_c_ and *Q*_c_ must be determined, *e.g.*, from thermal tuning of the cantilever. Amplitude *versus* distance curves must be acquired to evaluated the tapping mode set point amplitude *A*_sp_ in nanometers from the original electric signal. The core magnetization *M* must be known. Finally, *δ*_tip_, *i.e.*, the location of the magnetic dipole describing the probe, must be determined. While the curvature center of the apex could be the most natural choice, the non-spherical shape of the probe is expected to shift the position of the equivalent dipole. The determination of its actual position would require a more comprehensive investigation, *e.g.*, by simulating the magnetic field from the probe and fitting it with that of the dipole model. In an analogous investigation, which however was not extensive enough to allow us to extrapolate any general relation, a shift of about 10% of the location of the equivalent dipole from the curvature center of the apex was observed.^54^ Thus, the calibration proposed in this work still seems more advantageous.

Moreover, the validation of the method using different types of probes, *e.g.*, low moment (LM) or high moment (HM), would further demonstrate the generality of the approach. Really, the experiments reported in this work were conducted using probes that have medium/high moment and medium/high coercivity. These kinds of probes are the most widespread in MFM experiments as they are suitable for a broad range of magnetic samples and they offer a good signal-to-noise ratio. As far as the moment is concerned, LM probes are available, which are characterized by a moment that is a fraction (*e.g.*, 30%) of the moment of the probes used here. Thus, the detected magnetic signal is proportionally smaller, reducing the signal-to-noise ratio. In addition, while the magnetic field from NPs is low, in our experiments the lift height values vary from 20 nm to 150 nm, thus requiring probes also sensitive enough at large lift heights. LM magnetic probes seem, therefore, not promising for being used in our method. Conversely, HM probes are characterized by moment values up to 3 times larger than that of standard probes and, therefore, could be suitable for our method. From the point of view of the coercivity, the probes we used are characterized by a medium coercivity (nominal value 400 Oe), which are the most common and the most general-purpose ones. Significantly different values of coercivity characterize ultra-low coercivity probes (below 8 Oe), which, however, are not suitable for imaging magnetite NPs as their magnetic field can vary the magnetization of the tips. Conversely, high-coercivity probes could be suitable for our methodology. However, their use in our present experimental setup is prevented by the maximum magnetic field that our electromagnet can generate, which is not sufficient to fully magnetize the high coercivity probes.

Although commercial probes are the most widespread and general purpose in the scientific community, advanced probes have been proposed with well-defined geometries for quantitative and high-resolution MFM, albeit in only a limited number of papers. These advanced probes could improve the accuracy of our method not only regarding the lateral resolution, but for their geometry, which would simplify the modeling of the tip. Examples of these advanced magnetic probes are nanorod-shaped probes such as iron-filled carbon nanotubes,^85–87^ Co-coated carbon nanotubes,^88,89^ Co/Fe nanorods realised with focused electron-beam-induced deposition (FEBID).^90,91^ Also, the dipole–dipole model could be tested on probes realized attaching a single magnetic NP on a AFM tip.^92^ Actually, the use of such specialized probes is quite beyond the scope of the present work, which focuses on demonstrating the method with commercially available, widely accessible instrumentation. The calibration on bare NPs partially compensates for the imprecise knowledge of the tip geometry, absorbing uncertainties into the fitting parameters *A* and *x*_0_. However, the use of probes with well-defined geometry could in principle simplify the calibration procedure.

Notably, using only AFM to estimate the size of the NPs, in principle a rough estimation of the shell thickness can be obtained by measuring the diameters of a sufficiently large number of bare NPs in standard AFM morphologies and statistically evaluating their diameter *d*; then, for each core–shell NP, the outer diameter *D* is measured from AFM topographies and the shell thickness is evaluated as *c* = (*D* − *d*)/2. This approach, however, can be used only if the bare NPs have sufficiently uniform diameters, with a variability that is negligible with respect to the shell thickness. Indeed, in standard AFM, it would be impossible to retrieve information on the actual core diameter of a single core–shell NP. Conversely, in our method, the actual diameter of the core of the single core–shell NP does not need to be *a priori* assumed, but it is determined from the MFM curves. Therefore, the variability of the core diameter does not affect the uncertainty of the determination of the shell thickness. Moreover, as discussed above, strictly speaking our method requires only a one-time calibration. Therefore, after the initial calibration step (to evaluate at least *δ*_tip_), our method can also be used on core–shell NPs with a core material different from that of the calibration sample, providing the core magnetization is known. Thus, although our method is admittedly more time-consuming due to the initial need for calibration, it allows one to calculate the diameter of the core and the thickness of the shell of individual NPs selected on the surface without suffering from the uncertainty due to the assumption of an average core diameter.

## 5 Conclusion

In conclusion, a technique based on MFM for the measurement of the thickness of the nonmagnetic coating of core–shell magnetic NPs has been reported. The shell thickness is evaluated from the measured values of the magnetic phase shift, corrected for electrostatic artifacts, as a function of the lift height after calibration of the tip using uncoated NPs. The method was demonstrated using NPs with a magnetite core and a silica shell, which allowed us to compare the results obtained through MFM with direct inspection using TEM. Among the advantages, the proposed approach enables the characterization of specific NPs, which can be selected from AFM morphological images of the sample. Also, it is not limited by the actual material of the nonmagnetic coating and by its contrast with the material of the magnetic core. The technique is effective on isolated small NPs with a single magnetic core and a uniform shell. Conversely, in the case of small aggregates of NPs, it would supply effective values of core diameter and shell thickness. Despite this limitation, MFM is a promising tool in the investigation of core–shell magnetic NPs.

## Conflicts of interest

There are no conflicts to declare.

## Data Availability

Data for this article, including AFM and MFM data, are available at NOMAD at https://doi.org/10.17172/nomad.hx62-j6ss.
